# Correction: Feminization of the precarious at the UNAM: Examining obstacles to gender equality

**DOI:** 10.1371/journal.pone.0357363

**Published:** 2026-08-31

**Authors:** Lu Ciccia, Geraldine Espinosa-Lugo, Graciela Garcia-Guzman, Jaime Gasca-Pineda, Patricia Velez, Laura Espinosa-Asuar

There are errors in the legends of Fig 1 and 2. Please see the complete, correct Fig 1 and 2 legends here.

**Fig 1:**
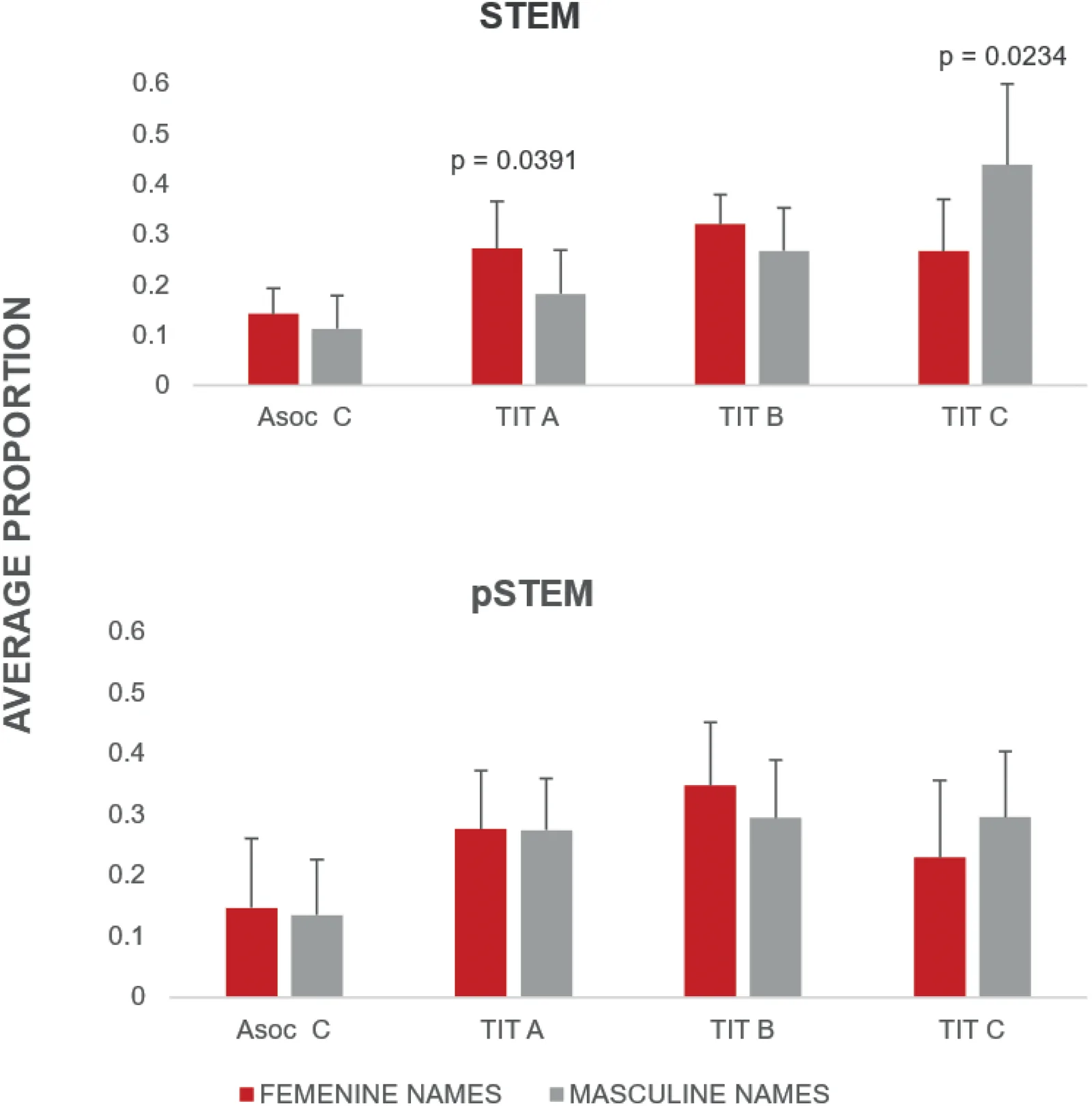
Average proportional distribution by gender among research personnel across academic rank categories. Statistically significant differences identified by the Wilcoxon rank-sum test are indicated.

**Fig 2:**
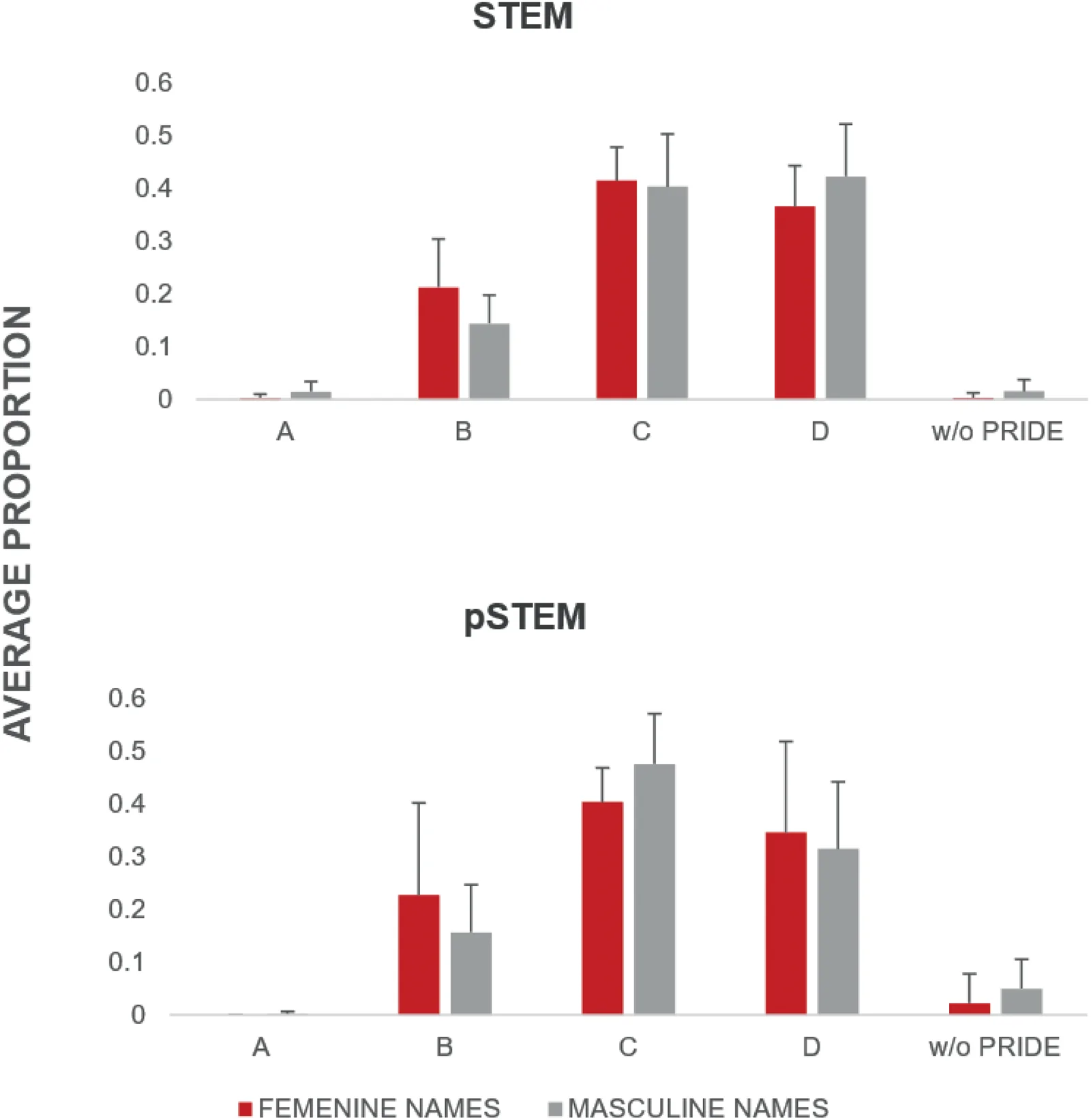
Average proportional distribution by gender among research personnel across PRIDE incentive program categories. No statistically significant differences were detected by the Wilcoxon rank-sum test.
