# Supplementary material for: Feminization of the precarious at the UNAM: Examining obstacles to gender equality
Source: PLoS One. 2025 Oct 29;20(10):e0334122. doi: 10.1371/journal.pone.0334122 (PMC12571264; doi:10.1371/journal.pone.0334122)
Supplement: S1 File — (DOCX) [file pone.0334122.s001.docx]

**Supplemental figures and tables**

ciccia *et al.* 2025

**Supplemental figures**

**
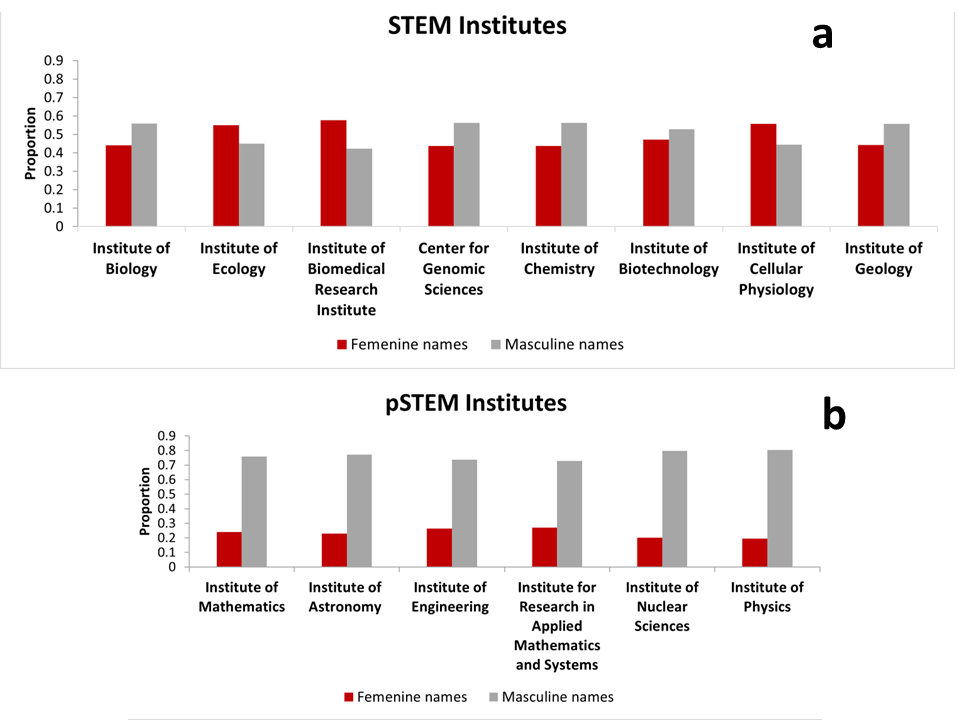
**

**Fig A. Percentage of masculine and feminine academic personnel names within (a) STEM and (b) pSTEM UNAM institutes.**

**
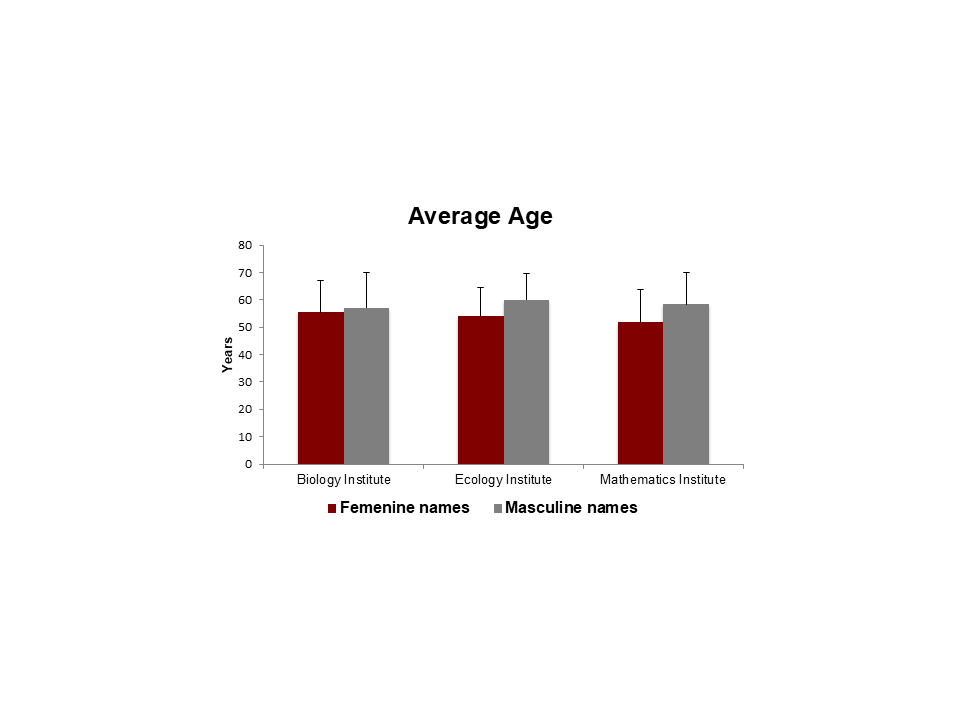
**

**Fig B. Age average and standard deviation of feminine and masculine research personnel in the three institutes.**

**
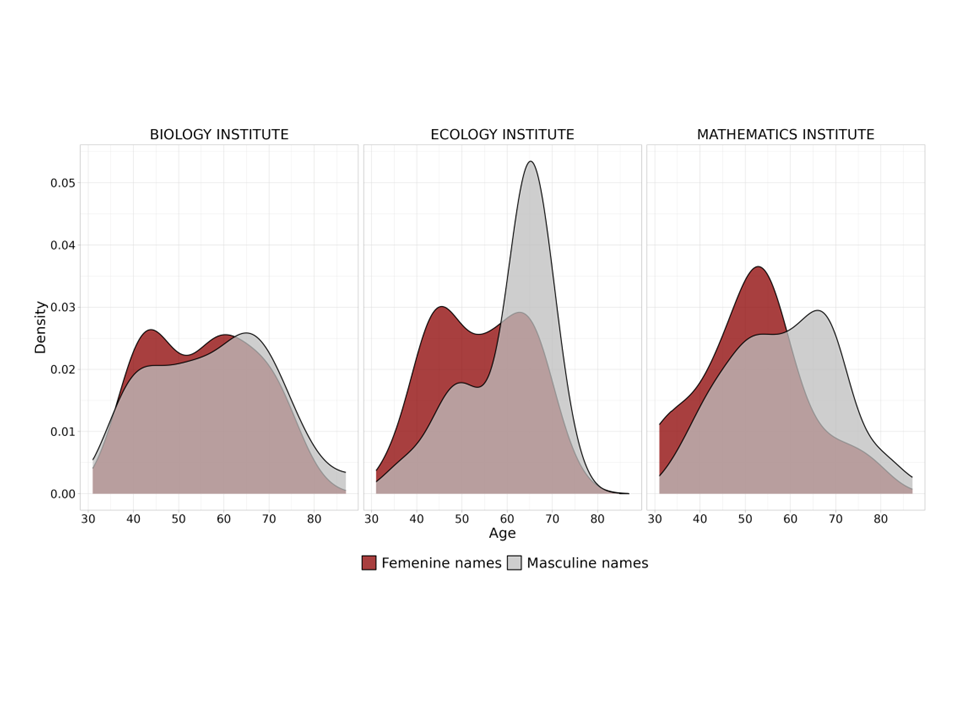
**

**Fig C. Age normal distribution by gender and institutes (research personnel data).**

**
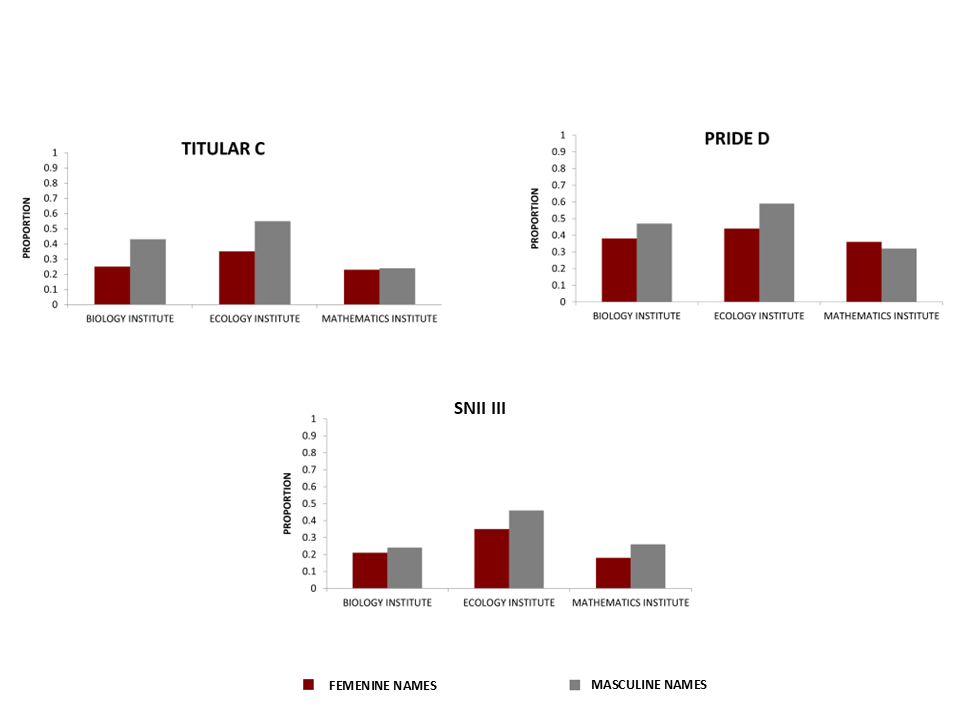
**

**Fig D. Proportional distribution comparison in the three UNAM institutes**. Comparison was made for the highest levels of academic ranks and incentive programs positions (feminine vs masculine research personnel), for the institutes of Biology, Ecology, and Mathematics.

**
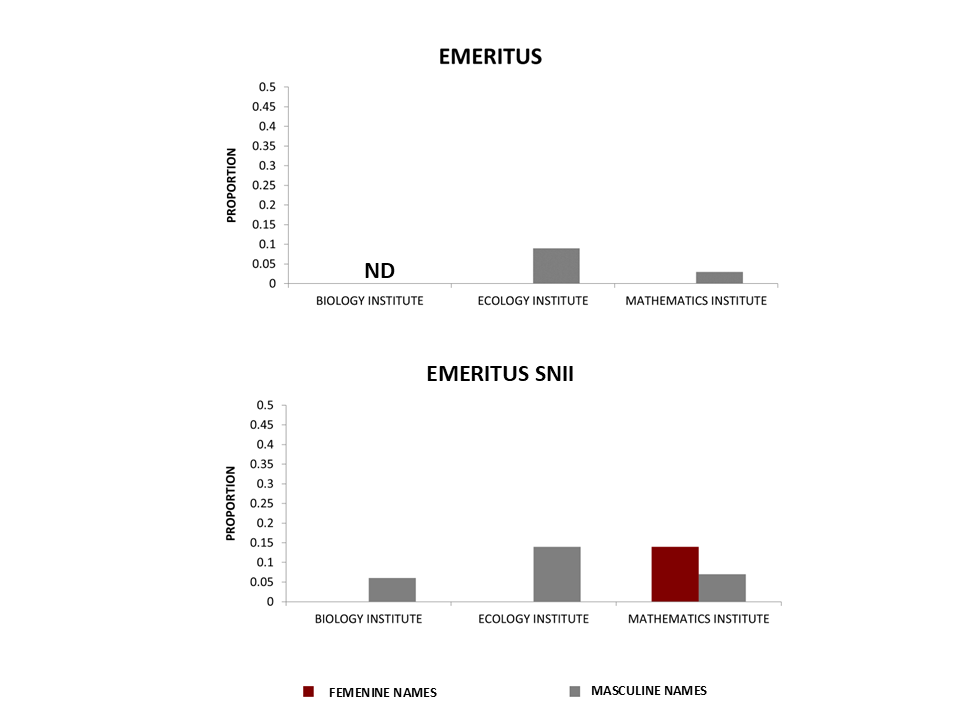
**

**Fig E. Proportional distribution of feminine and masculine research personnel occupying Emeritus distinction.** Both academic positions and SNII incentives within the three institutes are shown.

**
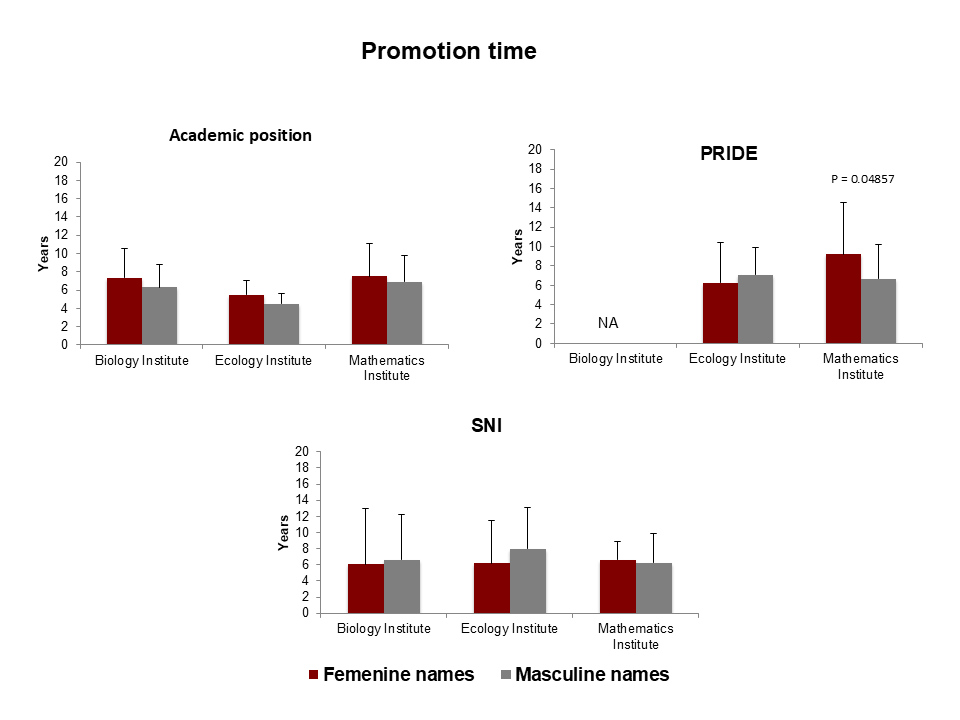
**

**Fig F. Average time and standard deviation for promotion (years) in feminine and masculine research personnel.** Academic (A) and incentive programs (B & C) positions within the 3 institutes analyzed (PRIDE IB data were not available). Significant comparisons show p-values.

**
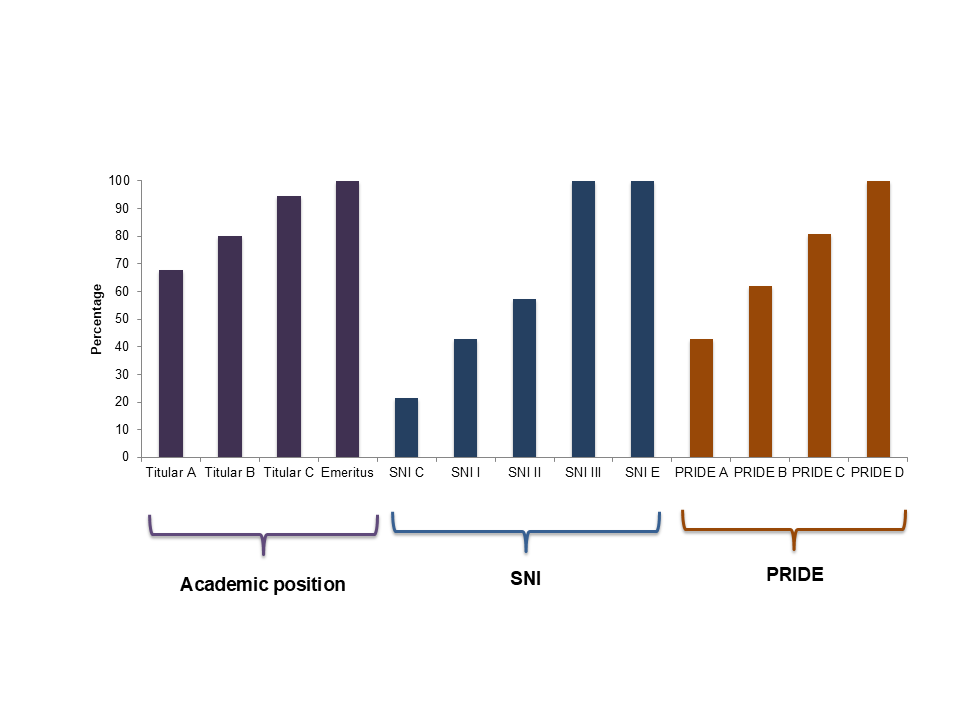
**

**Fig G. Remuneration differences.** Shown in percentage respect to the higher level within academic (research personnel data) and incentive programs positions (IP applies to all academic personnel).

**Supplemental tables**

**Table A. Feminine and masculine academic personnel count within the three institutes (IB, Institute of Biology; IE, Institute of Ecology; IM, Institute of Mathematics).**

| ACADEMIC PERSONNEL COUNTS | | | | | | |
| --- | --- | --- | --- | --- | --- | --- |
|  | IB | | IE | | IM | |
|  | F | M | F | M | F | M |
| Technical personnel | 47 | 39 | 21 | 14 | 6 | 15 |
| Research personnel | 24 | 51 | 23 | 22 | 22 | 73 |
| Total | 71 | 90 | 44 | 36 | 28 | 88 |

**Table B. Remuneration (Mexican pesos) by positions for the academic personnel of UNAM´s institutes corresponding to 2023.**

| POSITION | TECHNICAL PERSONNEL | RESEARCH PERSONNEL |
| --- | --- | --- |
| TITULAR A | 19,451.36 | 25,254.4 |
| TITULAR B | 21,882.76 | 29,851.24 |
| TITULAR C | 25,254.4 | 35,287 |
| EMERIT | NA | 37,265.66 |

The table is ordered from lower to higher levels. NA = Not Applicable (Emerit is a distinction that does not exist for technical personnel). For comparative purposes, we show only the three highest levels for technical personnel (those equivalent to research personnel levels).*

*Academic technical staff are responsible for both academic and technical tasks within the university, and there is an implicit devaluation of such roles. The association of ‘technical’ work with productive labour translates into the perception that ‘technical’ activities are of lower status, while academic activities are linked to greater prestige [26]. This devaluation is, of course, associated with economic precarity and can be observed when comparing the perceptions of academic technical staff with those of research staff.
