# Supplementary material for: Feminization of the precarious at the UNAM: Examining obstacles to gender equality
Source: PLoS One. 2025 Oct 29;20(10):e0334122. doi: 10.1371/journal.pone.0334122 (PMC12571264; doi:10.1371/journal.pone.0334122)
Supplement: S3 File — (DOCX) [file pone.0334122.s003.docx]

**Tables reporting p-values from Wilcoxon rank-sum tests**

ciccia *et al.* 2025

Two-sample paired Wilcoxon test for the standardized gender proportions in STEM and pSTEM disciplines for Academic positions. p-values are reported; values in bold denote significance.

|  | Ascoc. C | TC A | TC B | TC C |
| --- | --- | --- | --- | --- |
| pSTEM | 0.563 | 0.844 | 0.313 | 0.0625 |
| STEM | 0.547 | **0.0391** | 0.0547 | **0.0234** |

Two-sample paired Wilcoxon test for the standardized gender proportions in STEM and pSTEM disciplines for PRIDE levels. p-values are reported; values in bold denote significance.

|  | No PRIDE | A | B | C | D |
| --- | --- | --- | --- | --- | --- |
| pSTEM | 0.0591 | 0.999 | 0.313 | 0.219 | 0.563 |
| STEM | 0.181 | 0.100 | 0.781 | 0.844 | 0.250 |

Two-sample paired Wilcoxon test for gender differences in promotion times (Institutes of Biology, Ecology, and Mathematics). p-values are reported; values in bold denote significance.

|  | Academic positions | PRIDE | SNII |
| --- | --- | --- | --- |
| Institute of Biology | 0.3217 | NA | 0.2948 |
| Institute of Ecology | 0.2096 | 0.2853 | 0.1541 |
| Institute of Mathematics | 0.3831 | **0.04857** | 0.3965 |
