# Supplementary material for: Feminization of the precarious at the UNAM: Examining obstacles to gender equality
Source: PLoS One. 2025 Oct 29;20(10):e0334122. doi: 10.1371/journal.pone.0334122 (PMC12571264; doi:10.1371/journal.pone.0334122)
Supplement: Attachment — Submitted filename: Response to reviewers_cicciaetal_R1.docx [file pone.0334122.s005.docx]

**Response to reviewers**

**Manuscript: Feminization of the precarious at the UNAM: examining obstacles to gender equality**

**Authors: lu ciccia, Geraldine Espinosa-Lugo, Graciela García-Guzmán, Jaime Gasca-Pineda, Patricia Vélez, Laura Espinosa-Asuar**

**Journal Requirements**

When submitting your revision, we need you to address these additional requirements.

1. Please ensure that your manuscript meets PLOS ONE's style requirements, including those for file naming. The PLOS ONE style templates can be found at

https://journals.plos.org/plosone/s/file?id=wjVg/PLOSOne_formatting_sample_main_body.pdf and

https://journals.plos.org/plosone/s/file?id=ba62/PLOSOne_formatting_sample_title_authors_affiliations.pdf.

**R =** Done

2. We note that the grant information you provided in the ‘Funding Information’ and ‘Financial Disclosure’ sections do not match.

When you resubmit, please ensure that you provide the correct grant numbers for the awards you received for your study in the ‘Funding Information’ section.

**R=** Attended.

3. Thank you for stating the following financial disclosure:

[DGAPA, National Autonomous University of Mexico (UNAM) under grant PAPIIT IA400223].

Please state what role the funders took in the study. If the funders had no role, please state: ""The funders had no role in study design, data collection and analysis, decision to publish, or preparation of the manuscript."" If this statement is not correct you must amend it as needed. Please include this amended Role of Funder statement in your cover letter; we will change the online submission form on your behalf.

**R =** A new funding statement has been included in the cover letter.

4. Thank you for stating the following in the Acknowledgments Section of your manuscript:

[This work was supported by DGAPA, National Autonomous University of Mexico (UNAM) under grant PAPIIT IA400223. This grant PAPIIT IA400223 also funding scholarship of Geraldine Espinosa Lugo and Linda Gisele Zavala Hernández. We thank Nagapriya Wright for his impeccable translation. We appreciate the work of Camila Ramírez Araujo and Adriana Ruiz Gadea for collecting data.]

We note that you have provided funding information that is not currently declared in your Funding Statement. However, funding information should not appear in the Acknowledgments section or other areas of your manuscript. We will only publish funding information present in the Funding Statement section of the online submission form.

Please remove any funding-related text from the manuscript and let us know how you would like to update your Funding Statement. Currently, your Funding Statement reads as follows:

[DGAPA, National Autonomous University of Mexico (UNAM) under grant PAPIIT IA400223].

Please include your amended statements within your cover letter; we will change the online submission form on your behalf.

**R =** The funding statement has been updated, removed from the main text, and included in the cover letter.

5. Please expand the acronym “DGAPA” (as indicated in your financial disclosure) so that it states the name of your funders in full.

This information should be included in your cover letter; we will change the online submission form on your behalf.

**R =** We changed the funding statement as follows: This work was supported by Dirección General de Asuntos del Personal Académico, Universidad Nacional Autónoma de México (award number: PAPIIT IA400223; https://dgapa.unam.mx/index.php/impulso-a-la-investigacion/papiit). This grant also funded the scholarship of GEL. The funders had no role in study design, data collection and analysis, decision to publish, or preparation of the manuscript.

6. Please include captions for your Supporting Information files at the end of your manuscript, and update any in-text citations to match accordingly. Please see our Supporting Information guidelines for more information: <http://journals.plos.org/plosone/s/supporting-information>.

**R=** Done

**Additional Editor Comments**

- In your Methods section please clearly state how names were determined to be "masculine" or "feminine" and how edge instances were handled.

**R=** We appreciate the editor’s suggestion and have incorporated the requested clarification in the *Methods* section. We now explicitly state the procedure used to classify names as “masculine” or “feminine,” as well as the criteria applied to handle ambiguous or edge cases.

- While revising your submission, please upload your figure files to the Preflight Analysis and Conversion Engine (PACE) digital diagnostic tool, https://pacev2.apexcovantage.com/. PACE helps ensure that figures meet PLOS requirements. To use PACE, you must first register as a user. Registration is free. Then, login and navigate to the UPLOAD tab, where you will find detailed instructions on how to use the tool. If you encounter any issues or have any questions when using PACE, please email PLOS at figures@plos.org. Please note that Supporting Information files do not need this step.

**R =** We analyzed the figures in PACE as requested.

**Reviewers' comments**

**Reviewer #1**: This research explores an important topic, and the comparisons across STEM fields are valuable. The differences across discipline identified are interesting, and the explanation provided for those differences plausible. I would recommend some revisions to address some lingering questions and concerns.

1. First, and most importantly, I would recommend moving the methodology section from the end of the paper, so that it appears prior to the presentation of the results. It's actually hard to understand the findings without having the explanation of what data are being drawn on, and how they are being analysed. The findings will be more comprehensible if you describe what you did ahead of time.

**R =** We thank the reviewer for this valuable observation regarding the structure of the manuscript. Following this recommendation, we have relocated the *Methods* section so that it now precedes the *Results* section. This reordering ensures that readers are first presented with the data sources and analytical procedures, which we agree facilitates a clearer and more contextualized understanding of the findings.

2. Second, the methodology section can be expanded to provide explanations for key variables, so that people unfamiliar with academia in Mexico can better follow. For example, you provide a brief explanation of PRIDE and SN1, but it was not sufficient for me to understand what these programs are and, therefore, how to interpret gender differences with respect to them. Some aspects of the methodology are well-explained, but others are less explained.

**R =** We thank the reviewer for these helpful suggestions. We have added a section detailing the incentive programs and the organizational structure of academic staff at UNAM, as well as another section clarifying the procedure for classifying names as “masculine” or “feminine” and handling ambiguous cases. A new table has been included to illustrate the data collection process. Additionally, the *Methods* section has been reorganized to follow the division between quantitative data for all institutes and qualitative (descriptive) data from the three selected institutes. We hope these modifications provide clearer explanations that facilitate understanding of the key variables under study.

3. Third, you provide an explanation for how gender differences vary across discipline, but it wasn't clear that your data allowed you to test or support your explanation.

**R =** We highly appreciate this observation. Given that this is an interdisciplinary article, we recognize that the writing style and the interpretation of patterns from the perspective of feminist epistemology may be confusing when trying to identify trends from a more scientific point of view. We will address this reviewer’s comments in three parts, to respond carefully to each of the concerns raised.

3.1. Neither was it entirely clear that there was a robust body of literature that would support your conclusions, over other potential explanations for the differences. Did you have any measure for cultural capital, for example?

**R =** We carefully reviewed the interpretations and statements made from the perspective of feminist epistemology, providing more detailed references to the sources on which we relied and clarifying, where necessary, that these are suggestions to be tested in future research. As we recognize that alternative explanations may exist, we have added a paragraph to the manuscript to state this point: “*Finally, it is important to acknowledge that our interpretation of the observed pattern—namely, greater vertical segregation in STEM institutes compared to pSTEM institutes—may coexist with alternative explanations; nevertheless, we regard it as a reasonably accurate approximation, in line with the studies cited here and the critiques of gender biases from feminist epistemology*”.

We also clarify that *cultural capital* is a term we are putting forward: we specify that by *cultural capital* we refer to the social legitimacy (the prestige of the disciplines) and the economic remuneration associated with a given discipline, drawing on the framework proposed by Croft *et al.* 2015.

3.2. In the conclusion, you write, "Fields with high cultural capital create significant symbolic obstacles for women, whereas those with lower cultural capital impose structural challenges." It wasn't clear to me what this (and similar) statements are based on. Is there evidence of 'symbolic obstacles' or variations in cultural capital across fields? Why symbolic obstacles (versus discrimination or social closure for example)?

**R =** We agree that our original explanation was unclear. In the revised text, we clarify that, based on the patterns observed, we are offering suggestions that should be corroborated by future research. For the sake of clarity, we have removed this specific paragraph from the *Conclusions* section. Additionally, we have revised the text, particularly the conclusions to avoid definitive statements, and we have specified, where necessary, that these are suggestions to be tested. Finally, we have explicitly indicated in the text the references related to the requested evidence for symbolic barriers, as well as the variation of social legitimacy and economic remuneration (cultural capital) across fields (Croft *et al.* 2015, Fox 1985, Haraway 2005, Maffia 2005a, Maffia 2005b, Maffia 2007).

As mentioned above, we acknowledge that alternative explanations to our interpretation may exist, and this point is now made explicit in the text.

3.3. It seems like your data is primarily objective data on employment and recognition, with some ability to assess trends over time. However, there didn't seem to be any clear data that allowed you to understand why these differences occur. As a result, I am not convinced that your data entirely supports your conclusions.

**R =** As stated above, in the revised version, we have made explicit which patterns are directly supported by the data and which are our suggestions for future research. We hope this distinction clarifies the relationship between our data and the conclusions presented.

4. Fourth, you might want to also move the 'What is the underlying problem?' section. It is good to establish the 'problem' earlier in the paper. Here too, though, you should be careful not to declare that your findings support conclusions about structural and symbolic barriers, when it is not clear on what variables or analyses these conclusions are based. If you do have evidence of structural and symbolic barriers in academia, please be clearer about what these are.

**R =** After thinking carefully about this matter, we decided to keep the cited section at the end of the article, as it is specifically dedicated to proposing a possible solution (effective inclusion policies) to the biases observed; thus, presenting it at the beginning could be confusing. To clarify its purpose, we have changed the title of the section to “*De-gendering academic fields for equal opportunities.*”

We have also added a paragraph in the *Conclusions* section stating: *“We propose that vertical segregation arises from structural obstacles, such as gender-based discrimination, which limit women’s opportunities for professional advancement; however, this postulate requires further investigation.”*

Regarding the evidence for symbolic and structural barriers in academia, we have specified in the text relevant references referring to such barriers (Buquet 2013, Buquet *et al.* 2013, Maffia 2005a, Maffia 2005b, Maffia 2007).

**Reviewer #2**

1-All comparisons are presented descriptively (proportions and means) without significance testing. Incorporate appropriate tests such as chi-square for rank/incentive distributions, t-tests, or nonparametric equivalents for promotion intervals, and report p-values or confidence intervals to support claims about gender differences.

**R =** We thank the reviewer for this suggestion. We conducted statistical analyses on the data, which are now detailed in the *Methods* section. We updated the main figures accordingly to reflect these new analyses and reclassified some of the data as qualitative (descriptive). While these descriptive data support the observed pattern, the small sample size does not allow us to conclude that it is significant, a point explicitly stated in the text: “*Given the limited number of observations analyzed separately for each institute, we cannot conclude that the observed trend is significant, as evidenced by the statistical results (p > 0.05, data not shown). Nonetheless, using it as descriptive data is valuable because, on the one hand, it reflects the overall patterns observed across all institutes, and on the other, it includes information that is not widely available”.*

2- State explicitly whether this is a purely descriptive case-study or part of a mixed-methods design. If it is hypothesis‐driven, articulate the specific hypotheses being tested (e.g., “women in pSTEM will show shorter promotion intervals than women in STEM”).

**R =** We have made explicit throughout the text that this is a case study (although we have now included statistical analyses, so in some respects it has moved beyond the “purely descriptive”). We have also stated the hypothesis more clearly: *“We hypothesize that although there are few women in the pSTEM, they are less affected by vertical segregation than STEM women. To test this hypothesis, we analyzed vertical segregation regarding positions within academic and incentive programs at selected STEM and pSTEM institutes.”*

3-Provide a flowchart or table listing each data source, date accessed, query parameters (e.g., “all personnel records with active status as of July 1, 2023”), and any filters applied (e.g., exclusion of emeritus staff until 2018).

**R =** We appreciate this valuable suggestion to clarify how we obtained the data. As requested, we have included such a table in the *Methods* section.

4-By expanding the Methods to include precise search protocols, clear variable definitions, inferential and multivariable analyses, and transparent code/ethics statements, the authors will substantially improve the study’s credibility, reproducibility, and impact.

**R =** Thanks for the encouragement. In this new version, we have aimed to increase the reproducibility of the results (e.g., we have included a supplementary table containing the original data from all institutes), and hopefully, the new table incorporated in the *Methods* section will help clarify the data collection protocols. Throughout the text, we have included a more explicit definition of the variables. Also, the proposals and interpretations derived from feminist epistemology based on the data have been identified as such. In the case of this study, ethical declarations are not required, as it did not involve work at the personal level and all the data collected are publicly available.
